# Factors associated with a basic common drug-drug interaction knowledge among emergency department medical personnel

**DOI:** 10.1186/s40360-022-00623-0

**Published:** 2022-10-31

**Authors:** Theerapon Tangsuwanaruk, Borwon Wittayachamnankul

**Affiliations:** Department of Emergency Medicine, Faculty of Medicine, Chiang Mai University, 110 Inthawaroros Road, Sribhumi, Amphoe Muang Chiang Mai, Chiang Mai, 50200 Thailand

**Keywords:** Drug interactions, Education, Emergency Service, Hospital, Health Personnel, Pharmacology, Clinical

## Abstract

**Background:**

Drug-drug interactions (DDIs) are common but less concerning in clinical practice of time-sensitive situations. We aimed to identify factors associated with a basic common DDI knowledge among an emergency physician (EP), an emergency medicine resident (EMR), and an emergency care nurse (ECN).

**Methods:**

This was a prospective cross-sectional study. EP, EMR, and ECN did the examination (multiple-choice questions, 40 points) about common DDI. Prespecified factors associated with examination scores were profession, longer emergency medicine experience, pharmacological training, last advanced cardiovascular life support (ACLS) training, DDI checker book, and application user experience. The outcome was an examination score to evaluate the ability of DDI knowledge. Univariable and multivariable means regressions were used.

**Results:**

A total of 244 participants were enrolled. Factors associated with high examination score were EP (unadjusted mean difference 3.3 points, 95% confidence interval [CI] 2.1 to 4.5, p < 0.001), EMR (2.1, 95% CI 0.7 to 3.5, p 0.005) compared to ECN. Last ACLS training within 2 years (3.7, 95% CI 0.7 to 6.6, p 0.015), 2–4 years (3.4, 95% CI 0.4 to 6.5, p 0.027), and ≥4 years (4.4, 95% CI 1.2 to 7.6, p 0.007) were higher score than no ACLS training. Moreover, the DDI checker application experience user (1.7, 95% CI 0.6 to 2.8, p 0.003) also had a high score compared to the non-experienced user. After adjustment for all factors, EP (adjusted mean difference 3.3 points, 95% CI 1.8 to 4.7, p < 0.001), EMR (2.5, 95% CI 0.6 to 4.3, p 0.010) were higher scores compared to ECN. Meanwhile, the last ACLS training ≥4 years (3.3, 95% CI 0.1 to 6.6, p 0.042) was a higher score than no ACLS training.

**Conclusion:**

EP, EMR, and the last ACLS training ≥4 years were associated with higher DDI knowledge than ECN and no ACLS training, respectively.

**Supplementary Information:**

The online version contains supplementary material available at 10.1186/s40360-022-00623-0.

## Introduction

Drug-drug interactions (DDI) are common clinical problem and can occur in several different ways, including pharmacokinetic and pharmacodynamics interactions.[1–4] In 2020, the American Association of Poison Control Centers reported 18,988 DDI information requests and 3,541 scenarios for therapeutic errors of medical personnel caused by DDI.[5] The incidence of DDIs is even more common in emergencies where the window period is narrow and rapid access to therapy is essential.[6] A recent observational study found up to 38% of prescriptions written upon discharge from the Emergency Department (ED) have at least one DDI.[7] Several previous studies also reported high DDI in patients with polypharmacy.[1, 8–13] Polypharmacy is common in elderly patients due to treating previously multiple medical conditions. More than half of the hospitalized elderly patients are exposed to at least one potential DDI, while one-fifth suffers from at least one potentially severe DDI.[14] Also, the elderly patient is more at risk of having a potential DDI than another age group in the ED due to polypharmacy.[6] Pediatric patients who visited the ED also suffer from DDIs, around 15% of which are severe.[15].

In the ED, medical personnel often face time-sensitive situations, and they may prescribe drugs to the patient before a formal prescription is executed on the computer. This practice bypasses the automatic DDI checker software in the computer-based prescribing system, which intends to help reduce basic common DDIs. Moreover, some resource-limited EDs were no pharmacists available; they stored the emergency medication on their local shelves for emergency patients. In this setting, pharmacists in the central pharmacy department in the hospital could not recheck before the physicians used those medications for their patients. For this reason, basic common DDI knowledge among ED personnel is, thus, of particular importance. However, few studies investigate the knowledge of emergency personnel. Identification of factors associated with that knowledge could be used to tackle this problem and plan for future ED training in this regard.

Our research question was which of the factors associated with a basic common drug-drug interaction (DDI) knowledge among an emergency physician (EP), an emergency medicine resident (EMR), and an emergency care nurse (ECN). Therefore, the objective of this study was to identify the factors associated with basic common DDI knowledge among ED medical personnel, including EP, EMR, and ECN.

## Methods

### Study design and setting

This prospective cross-sectional study was conducted in Thailand between June 11 and September 30, 2020. The Research Ethical Committee of the Faculty of Medicine, Chiang Mai University (No. 197/2020 on June 8, 2020) approved this research protocol. We obtained informed consent from all participants in the study. We followed the strengthening the reporting of observational studies in epidemiology (STROBE) statement recommendations.[16] The study was carried out in accordance with ethical guidelines of the Faculty of Medicine, Chiang Mai University. We prospectively registered this study on the Thai Clinical Trials Registry (TCTR20200610005) on June 10, 2020. We have complied with the latest version of the Declaration of Helsinki (2013).

### Study population

EPs, EMRs, or ECNs who worked in a hospital in Thailand were invited to participate in this study voluntarily. ECNs were registered nurses (RN), emergency nurses (EN), and emergency nurse practitioners (ENP). RNs, ENs, and ENPs were nurses who cared for the patients in the ED. Moreover, RNs passed a basic emergency medicine short course. ENs passed an intermediate emergency medicine course. ENPs were a specialist emergency nurses who passed a full emergency medicine course for the nurse.

Nurses have generally studied clinical pharmacology emphasizing drug preparation and administration to a patient. Meanwhile, physicians have studied emphasizing the indication and contraindication of each drug for specific diseases. Because ECNs are inevitably involved in drug preparation and administration, ECNs were enrolled in this study to determine the ability of DDI knowledge. In some emergencies, physicians may focus on indications of drugs given to their patients leading to less awareness of the risk of DDI. Although nurses cannot authorize which drugs should be prescribed to their patients, the ECN could be a key person to alert physicians of potential DDIs in prescribing drugs, especially in time-sensitive emergency circumstances.

### Study conduct and data collection

An invitation message was sent through social media, such as a specific social media group of emergency personnel. Recruitment via social media and an online survey platform was used because this method could effectively enroll personnel anywhere in Thailand. Moreover, the participant’s convenience in responding to this survey could encourage them to complete it more than the paper-based method. The study was introduced as follows: “Importance: This research participant is only for an emergency physician, an emergency medicine resident, or an emergency care nurse. If you are out of the scope of this profession, please send this survey to those you know.“ in an invitation message to ensure the participant was the target profession. Participants gave informed consent and answered the questionnaire through a secure internet-based examination survey using the Research Electronic Data Capture (REDCap) platform.[17].

The survey consisted of baseline characteristics (including potential factors) and multiple-choice questions (MCQs; 40 points) of DDI. If the survey was performed multiple times by one individual, only the score from the first time would be used for analysis. The score was sent to each participant via email after the data collection period.

Baseline characteristics included age, sex, academic degree, academic position, academic year of EMR, ECN level, emergency medicine experience, clinically pharmacological or toxicological training (including the time since the last completion or recertification of the clinically pharmacological or toxicological training), the advanced cardiovascular life support (ACLS) training (including the time since the last ACLS training), prior experience of DDI checker book and application use.

Prespecified factors in primary and secondary outcomes were profession (EP, EMR, or ECN), emergency medicine experience, clinically pharmacological or toxicological training, last completed or recertified ACLS training, prior experience of DDI checker book, and application use. These factors were planned to use in adjusting in multivariable analysis.

In ACLS course, some emergency drugs and their DDIs were taught in this course. For example, adenosine is prescribed to terminate supraventricular tachycardia, which was taught in the ACLS course. Patients concurrently using theophylline may diminish adenosine’s therapeutic effect as DDI, and a higher adenosine dose may be required to achieve its therapeutic effect. Therefore, medical personnel who recently passed the ACLS course might remember this DDI.

A DDI checker book may be available in a medical textbook, such as internal medicine, clinically pharmacological or toxicological textbook. Nowadays, a DDI application or web-based checker is widely available. However, the emergency personnel had a variety of generations of education. Some of them had utilized the early version of the DDI checker available in the platform as a book before it became an application or web-based tool. This study’s objective was to include various generations of emergency personnel; therefore, DDI checker book use experience was included as a factor even though currently DDI is used as an application or web-based checker.

The DDI questions were designed based on the classification of DDIs from the Lexicomp® Drug Interactions database from Lexi-Interact®.[18] DDI risk rating was classified into five levels: risk rating X (avoid combination), risk rating D (consider therapy modification), risk rating C (monitor therapy), risk rating B (no action needed), and risk rating A (no known interaction). Forty pairs of drugs were selected based on the chance to be used concomitantly (eight pairs with risk rating X, seven pairs with risk rating D, five pairs with risk rating C, and twenty pairs with risk ratings B or A). The correct answer is worth 1 point each, making the total score 40 points. The proportion of questions regarding risk ratings B or A to risk ratings X, D, or C was designed to be 1:1 (20: [8 + 7 + 5] = 20:20). The reason for grouping all risk ratings into three choices in MCQ was easy to answer and persuasion to complete all 40 MCQs. Internet-based examination survey in this study was available in the supplementary material.

### Outcome measures

The primary outcome was the examination score of all-risk ratings of common DDI from MCQs of the DDI questions. Secondary outcomes focused on examination scores of risk ratings X, D, and C. Moreover, we planned to identify an academic year of EMR (the first, second, and third residency years) associated with knowledge of all-risk rating DDI as a subgroup analysis.

### Sample size estimation

In biostatistics literature, a sample size of approximately 30 participants per variable has better power to detect a small effect size in regression analysis.[19] In our study, the sample size was also estimated by the formula as 30 participants per factor. With six prespecified factors, a sample size of 180 participants was estimated. To achieve 20% missing or incomplete data, a sample size of 225 participants was planned for enrollment.

### Data analysis

Descriptive data were presented as a number, percentage, mean, median, standard deviation, and interquartile range as appropriate. Categorical data were compared by the chi-squared test or Fisher’s exact test, as appropriate. Continuous data were compared by one-way analysis of variance (ANOVA) or the Kruskal-Wallis test, as appropriate. The Shapiro-Wilk test and data visualization was used to determine normal distribution. Missing data were planned to handle by the multiple imputation method. Univariable (unadjusted) and multivariable (adjusted) regression analyses of the means were used to determine the association between characteristics, prespecified factors and the knowledge score. The Stata version 16 (Stata Corp LLC, College Station, Texas, USA) was used for statistical analysis. Statistical significance was determined at p < 0.05.

## Results

**Fig. 1:**
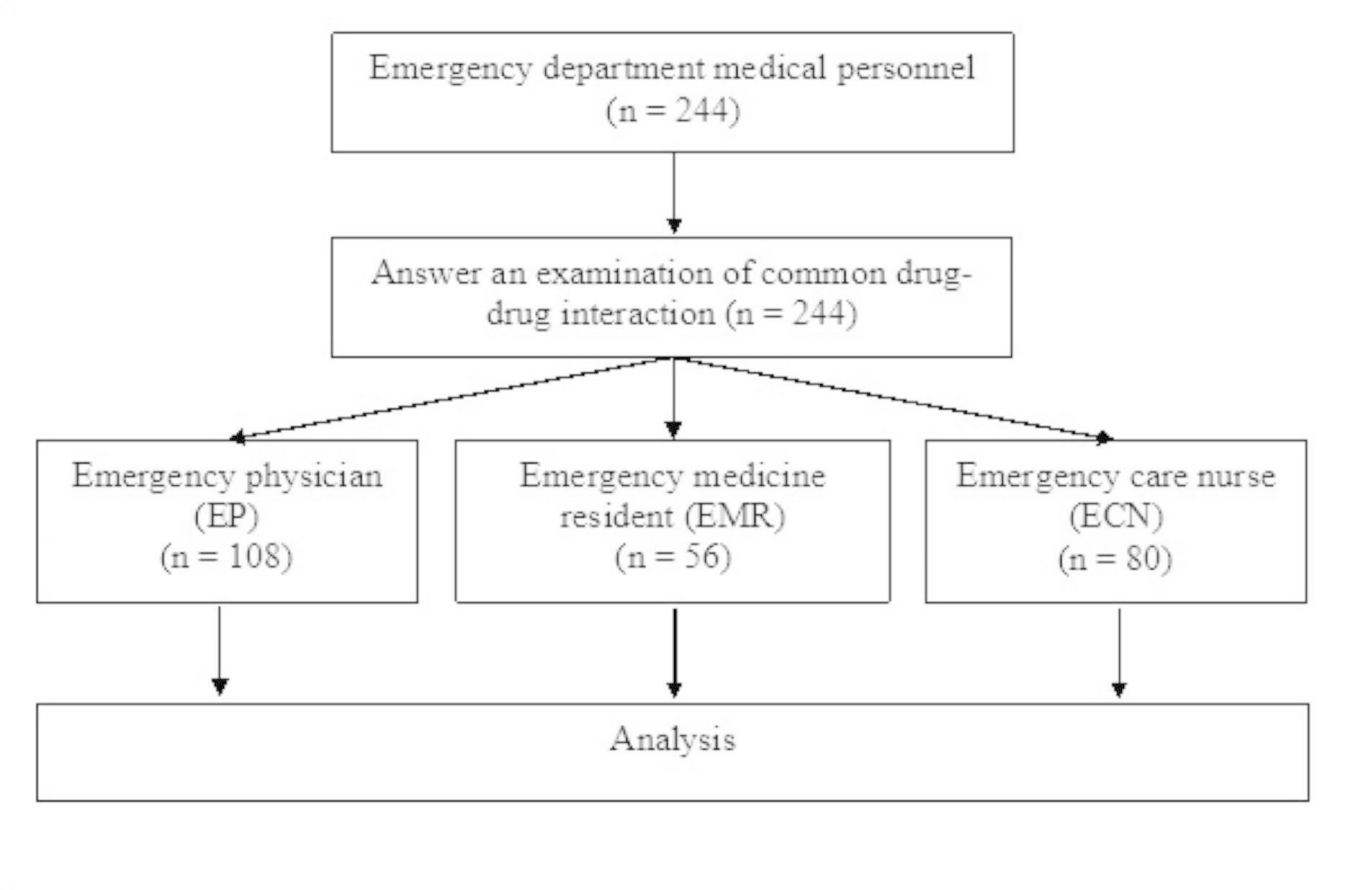
Study flowchart

A total of 244 participants were enrolled as 108 EPs (44.3%), 56 EMRs (23%), and 80 ECNs (32.7%) (Fig. 1). Most of them were females (62%), with a bachelor’s degree (84%), and no academic position (86.9%). All baseline characteristics differed among EP, EMR, and ECN except for the number of years of last completed or recertified ACLS training and the DDI checker book use experience (Table 1). There were no missing data or incomplete surveys.

**Table 1:** Baseline characteristics

Characteristics	Overall (n = 244)	Emergency physician (n = 108)	Emergency medicine resident (n = 56)	Emergency care nurse (n = 80)	p-value*
Age – year ^a^	32 (28, 36)	34 (32, 37)	28 (27, 30)	31 (26, 37)	< 0.001
Female – n (%) ^b^	152 (62)	62 (57)	30 (54)	60 (75)	0.014
Academic degree – n (%) ^b^					
Bachelor’s Degree	204 (84)	79 (73)	55 (98)	70 (88)	< 0.001
Master’s Degree	32 (13)	21 (19)	1 (2)	10 (12)	
Doctoral Degree	8 (3)	8 (8)	0	0	
Academic position – n (%)^b^					
None	212 (86.9)	77 (71.3)	56 (100)	79 (99)	< 0.001
Lecturer	29 (11.9)	29 (26.9)	0	0	
Assistant professor	2 (0.8)	2 (1.9)	0	0	
Associate professor	1 (0.4)	0	0	1 (1)	
Academic year of an emergency medicine resident – n (%) ^b^					
First-year	-	-	17 (30.4)	-	< 0.001
Second-year	-	-	14 (25)	-	
Third-year	-	-	25 (44.6)	-	
Emergency care nurse level – n (%) ^b^					
Registered nurse (RN)	-	-	-	39 (49)	< 0.001
Emergency nurse practitioner (ENP)	-	-	-	32 (40)	
Emergency nurse (EN)	-	-	-	9 (11)	
Emergency medicine experience – year ^a^	4 (2, 8)	5.5 (3.5, 10)	2 (1, 3)	7 (3, 11)	< 0.001
Clinically pharmacological or toxicological training – n (%) ^b^	58 (23.8)	34 (31.5)	19 (33.9)	5 (6.3)	< 0.001
Last completed or recertified clinically pharmacological or toxicological training – year ^a, c^	2 (1, 3)	2 (1, 4)	1 (1, 1)	3 (2, 6)	< 0.001
ACLS training – n (%) ^b^	235 (96.3)	108 (100)	55 (98.2)	72 (90)	0.001
Last completed or recertified ACLS training – year ^a, d^	1 (1, 3)	1.5 (1, 3)	1 (1, 2)	1 (1, 3.5)	0.051
Drug-drug interaction checker book use experience – n (%) ^b^	74 (30.3)	37 (34.3)	12 (21.4)	25 (31)	0.232
Drug-drug interaction checker application use experience – n (%) ^b^	97 (39.8)	60 (55.6)	30 (53.6)	7 (8.8)	< 0.001

Abbreviation: ACLS, advanced cardiovascular life support

* p-value < 0.05 is statistically significant

^a^ Median (interquartile range) and the difference between groups were analyzed by Kruskal–Wallis test

^b^ Differences between groups were analyzed by Fisher’s exact test

^c^ Only participants who pass the clinically pharmacological or toxicological training were included in the analysis

^d^ Only participants who pass the ACLS training were included for analysis

For the primary outcome of all risk ratings, it was found that EP and EMR had an examination score higher than ECN (Table 2). No evidence of years of emergency medicine experience and passing clinically pharmacological or toxicological training affected the score. Participants who had previously completed or recertified ACLS training for ≥ 4 years had the highest score compared to participants who had never passed ACLS training before. There was no evidence that previous DDI checker book use experience affected the score (19.6 ± 3.9 points in the user group compared to 18.6 ± 4.6 points in the never use group, an unadjusted mean difference of 1 point, 95% CI -0.2 to 2.2 points, p 0.108). Participants who used DDI checker application had a higher score than those who never used it (19.9 ± 3.8 points in the user group compared to 18.2 ± 4.6 points in the never use group, an unadjusted mean difference of 1.7 points, 95% CI 0.6 to 2.8 points, p 0.003).

**Table 2:** Primary outcomes: examination score of all-risk ratings of common drug-drug interactions

Variables	Examination score of all-risk ratings (total of 40 points) ^a^	Unadjusted		Adjusted	
		**Mean difference (95% CI)**	p-value*	**Mean difference (95% CI)**	p-value*
Profession					
Emergency physician (EP)	20.3 ± 3.9	3.3 (2.1 to 4.5)	< 0.001	3.3 (1.8 to 4.7)	< 0.001
Emergency medicine resident (EMR)	19 ± 4.3	2.1 (0.7 to 3.5)	0.005	2.5 (0.6 to 4.3)	0.010
Emergency care nurse (ECN)	17 ± 4.4	Ref		Ref	
Emergency medicine experience					
< 2 years	18.9 ± 4.3	Ref		Ref	
2–4 years	18.6 ± 4.5	-0.3 (-2.1 to 1.5)	0.743	0.1 (-1.7 to 1.8)	0.933
≥ 4 years	19.1 ± 4.4	0.2 (-1.4 to 1.8)	0.808	0.2 (-1.7 to 2)	0.864
Clinically pharmacological or toxicological training					
Never training	19 ± 4.2	Ref		Ref	
Ever training	18.7 ± 4.9	-0.2 (-1.6 to 1.1)	0.713	-1.3 (-2.6 to 0.1)	0.056
Last completed or recertified ACLS training					
Never training	15.3 ± 4	Ref		Ref	
< 2 years	19 ± 4.1	3.7 (0.7 to 6.6)	0.015	2.2 (-0.8 to 5.2)	0.153
2–4 years	18.8 ± 4.6	3.4 (0.4 to 6.5)	0.027	2 (-1.1 to 5.2)	0.203
≥ 4 years	19.7 ± 4.8	4.4 (1.2 to 7.6)	0.007	3.3 (0.1 to 6.6)	0.042
Drug-drug interaction checker book use experience					
Never use	18.6 ± 4.6	Ref		Ref	
Ever use	19.6 ± 3.9	1 (-0.2 to 2.2)	0.108	1 (-0.2 to 2.2)	0.116
Drug-drug interaction checker application use experience					
Never use	18.2 ± 4.6	Ref		Ref	
Ever use	19.9 ± 3.8	1.7 (0.6 to 2.8)	0.003	0.5 (-0.8 to 1.7)	0.470

Abbreviations: ACLS, advanced cardiovascular life support; Ref, reference; 95% CI, 95% confidence interval

* p-value < 0.05 is statistically significant

^a^ Mean ± standard deviation and mean difference between groups were analyzed by regression analysis of means

After using the multivariable regression, EP and EMR still were higher scores than ECN. Participants who had previously completed or recertified ACLS training for ≥ 4 years still had higher scores than participants who never had. No evidence of years of emergency medicine experience, previous clinically pharmacological or toxicological training, previous DDI checker book, or application use affected the score.

For the secondary outcome of risk ratings X, D, and C, it was found that no evidence of profession, emergency medicine experience, previously clinically pharmacological or toxicological training, last ACLS training, previously used DDI checker book, and application affected the score (Table 3). After adjusting the influence of other independent variables by using the multivariable regression, the results were unchanged.

**Table 3:** Secondary outcomes: examination score of risk ratings X, D, and C of common drug-drug interactions

Variables	Examination score of risk ratings X, D and C (total of 20 points) ^a^	Unadjusted		Adjusted	
		**Mean difference (95% CI)**	p-value*	**Mean difference (95% CI)**	p-value*
Profession					
Emergency physician (EP)	6.7 ± 2.3	-0.2 (-0.9 to 0.5)	0.571	-0.3 (-1.1 to 0.6)	0.516
Emergency medicine resident (EMR)	6.3 ± 2.1	-0.6 (-1.5 to 0.2)	0.133	-0.9 (-2.0 to 0.2)	0.117
Emergency care nurse (ECN)	6.9 ± 2.7	Ref		Ref	
Emergency medicine experience					
< 2 years	7.0 ± 1.8	Ref		Ref	
2–4 years	6.5 ± 2.4	-0.5 (-1.5 to 0.5)	0.332	-0.7 (-1.8 to 0.3)	0.155
≥ 4 years	6.7 ± 2.5	-0.3 (-1.2 to 0.6)	0.558	-0.8 (-1.9 to 0.3)	0.147
Clinically pharmacological or toxicological training					
Never training	6.8 ± 2.4	Ref		Ref	
Ever training	6.4 ± 2.3	-0.4 (-1.2 to 0.3)	0.221	-0.3 (-1.1 to 0.4)	0.367
Last completed or recertified ACLS training					
Never training	6.8 ± 3.7	Ref		Ref	
< 2 years	6.6 ± 2.2	-0.2 (-1.8 to 1.4)	0.796	0.4 (-1.4 to 2.1)	0.662
2–4 years	6.8 ± 2.3	0.1 (-1.6 to 1.7)	0.950	0.9 (-1.0 to 2.7)	0.349
≥ 4 years	6.8 ± 2.7	0 (-1.8 to 1.7)	0.987	0.5 (-1.3 to 2.4)	0.584
Drug-drug interaction checker book use experience					
Never use	6.5 ± 2.4	Ref		Ref	
Ever use	7.0 ± 2.3	0.5 (-0.2 to 1.1)	0.168	0.5 (-0.2 to 1.2)	0.161
Drug-drug interaction checker application use experience					
Never use	6.7 ± 2.5	Ref		Ref	
Ever use	6.7 ± 2.3	0.1 (-0.6 to 0.7)	0.827	0.1 (-0.6 to 0.9)	0.706

Abbreviations: ACLS, advanced cardiovascular life support; Ref, reference; 95% CI, 95% confidence interval

* p-value < 0.05 is statistically significant

^a^ Mean ± standard deviation and mean difference between groups were analyzed by regression analysis of means

Subgroup analysis showed the academic year of EMR was not associated with scores (Table 4). The first, the second, and the third academic year of EMR had the examination score of all-risk rating DDI as follows 18.8 ± 3.5, 20.9 ± 3.9, and 18.2 ± 4.8 points, respectively. The mean difference between the second and first years of EMR was 2.1 points (95% CI -1 to 5.2 points, p 0.177). The mean difference between the third and first years of EMR was − 0.6 points (95% CI -3.2 to 2.1 points, p 0.674).

**Table 4:** Secondary outcomes: subgroup analysis of an academic year of emergency medicine residency associated with examination scores

Variables	Examination score of all-risk ratings (total of 40 points) ^a^	Unadjusted	
		**Mean difference** **(95% CI)**	p-value*
Academic year of an emergency medicine resident (n = 56)			
First year (n = 17)	18.8 ± 3.5	Ref	
Second year (n = 14) ^b^	20.9 ± 3.9	2.1 (-1 to 5.2)	0.177
Third year (n = 25) ^c^	18.2 ± 4.8	-0.6 (-3.2 to 2.1)	0.674

Abbreviations: Ref, reference; 95% CI, 95% confidence interval

* p-value < 0.05 is statistically significant

^a^ Mean ± standard deviation and mean difference between groups were analyzed by regression analysis of means

^b^ Power-back calculations between the first year and the second year emergency medicine resident were analyzed and showed power as 0.33

^c^ Power-back calculations between the first year and the third year emergency medicine resident were analyzed and showed power as 0.07

As we need to demonstrate which profession tended to answer with a higher risk rating than the correct answer, a post hoc analysis was conducted and found that ECN answered higher risk ratings than EP (unadjusted mean difference 4.4 items, 95% CI 2.7 to 6 items, p < 0.001). There was no difference between EMR and EP (unadjusted mean difference 0.9 items, 95% CI -0.9 to 2.8 items, p 0.314).

A post hoc analysis was performed in the subgroup of EP and EMR to demonstrate the score between them of all risk ratings. In univariable analysis, there was no difference in the score between EP (20.3 ± 3.9 points) and EMR (19 ± 4.3 points) (unadjusted mean difference 1.3 points, 95% CI -0.1 to 2.6 points, p 0.062). Also, there was no difference in the score in multivariable analysis (adjusted mean difference 0.4 points, 95% CI -1.5 to 2.2 points, p 0.685).

## Discussion

Our study found that influencing factors associated with basic common DDI knowledge among ED medical personnel were EP, EMR, and the last ACLS training ≥4 years.

Ko et al. also reported that 42.7% of all drug combinations were classified as DDI correctly by the prescriber.[20] Similarly, our study found that EP had an average score for all risk ratings of 20.3 points out of a total of 40 points (51%). Although EP, and EMR had a higher examination score than ECN, they had an examination score of about half of the total score. It might be a major health service problem concerning emergency medical personnel competency and might lead to an adverse event from potential DDIs. Therefore, we encourage setting up a course to train about common DDI for the emergency medical personnel similar to other essential life support courses.

In the case of overall knowledge of all risk ratings of common DDIs, we found that EP, and EMR had a higher score than ECN. Our result differs from Ko et al., which found that physician and nurse practice did not affect DDI knowledge.[20] Warholak et al. found that medical students and nurse students had no difference in the ability of DDIs.[21] However, the Ko et al. study participant was a physician and nurse that was not subclassed as EP and ECN.[20] Warholak et al. studied medical and nurse students; therefore, those participants might differ from our participants.[21].

On the other hand, no evidence of difference among EP, EMR, and ECN in risk ratings X, D, and C. Physicians may focus on indications in emergency conditions of patients without awareness of drug pairs at risk of moderate to severe DDIs. While for ECNs, those involved in drug preparation and administration may be aware of drugs with moderate to severe DDIs, as well as high-alert drugs. However, no study demonstrated that physicians or nurses were more aware of DDI. Therefore, for the training of DDIs to physicians and nurses, there should be an emphasis on DDI at risk ratings X, D, and C.

Another hypothesis was that the ECN tended to answer drug pairs as higher risk ratings leading to a higher score. From our post-hoc analysis, ECN tended to answer with a higher risk rating than EP. However, this could be a practical advantage. When a nurse was unsure or mentioned as having a higher risk rating, a DDI checker may be used or alert the physician to recheck for DDIs.

The DDI knowledge between EP and EMR was not different in our post hoc analysis. Similarly, Yuan et al. conducted an online DDI survey focused on physicians and reported that physicians had an unsatisfactory knowledge level about clinically significant DDI (correctly classified 33.4% of DDIs).[22] Also, Nabovati et al. found that medical residents of 22 specialties correctly classified the DDI as only 41%.[23] Thus, medical education about DDI since undergraduate degree and residency training could be essential, including introducing an accessible electronic DDI database that might help physicians recognize DDI before prescribing medication to their patients.

A pharmacist, one of the specialists integrated into multidisciplinary teams, might be crucial in decreasing DDI incidents and can contribute to the DDI training of emergency professionals. If possible, we suggest caring for all patients in the ED with a multidisciplinary team, including a pharmacist combined with DDI screening software.[24] If the pharmacist recognizes any risk ratings of DDIs, the emergency physician and the nurse in the ED should be informed to reconsider the concurrent medications prescribed to the patient. However, the pharmacist might not always be available in the ED, such as in small hospitals or some hospitals in a developing country. This circumstance is a limitation of the ED in our hospital.

The number of years of emergency medicine experience did not affect knowledge. Even though working in the ED for a long time still unawareness of the DDI did not impact the score. Ko et al. also found that experience in prescribing medicines did not affect DDI knowledge.[20] Therefore, all ED personnel should attend this training regardless of their years of emergency medicine experience.

Most of clinically pharmacological or toxicological training in which emergency personnel participated were short-course style, teaching regarding drug overdose or substance abuse rather than DDIs.[25] Therefore, previous clinically pharmacological or toxicological training did not influenced DDI knowledge in our study. Owing to the importance of DDI knowledge, we encourage adding the DDI class into the clinically pharmacological or toxicological short course training.

In cases of the univariable analysis of all risk ratings, we found that the tendency of the longer last ACLS training, the higher score. However, in the analysis with the multivariable analysis to adjust for other factors, including emergency medicine experience, the only participant with the last ACLS training ≥ 4 years was related to a higher score. We believe that this could be from more experience in other medical fields regardless of emergency medicine experience. Further study might be conducted to explore the effect of subspecialty or other medical areas on ED personnel. There are two types of certificates for ACLS courses: ACLS provider (student certificate) and ACLS instructor certificate. The ACLS provider certificate should be renewed every two years. In general, ACLS instructors were certified as ACLS providers several times for several years. In addition, they would not require completing formal ACLS provider courses again because teaching ACLS provider courses within specific requirement numbers could be enough for automatic renewal. As a result of the overlapping of participants who have prior last ACLS provider training ≥ 4 years and participants who have ACLS instructor certificate, ACLS instructor might be answered (in our survey) the last ACLS training as their last ACLS provider instead of the last teaching in ACLS provider course. Therefore, experience in ACLS (ACLS instructor) might affect the DDIs knowledge. As our limitation, we could not differentiate their last status whether ACLS provider (need to participate in formal ACLS provider renewal course every two years) or ACLS instructor (unnecessary to participate in formal ACLS provider renewal course every two years). Further study should address this issue.

In the case of risk ratings X, D, and C, the last ACLS training did not affect the score. All of this may result from the ACLS training course not providing in-depth content on DDI topics, especially those with high-risk DDIs.

DDI checker book and application user experience did not affect the score whether all risk ratings or risk ratings X, D, and C by multivariable analysis. We believe that there are many DDIs in the ED making it difficult to remember to use them in a later decision in clinical practice. Although they can remember the results of having DDIs, they may not remember which risk rating it is.

Subgroup analysis of the academic year of EMR was not associated with examination scores, which may result from EMR training does not emphasize DDI training. However, when performing a post-hoc power-back calculation, it was found that the low power of statistics to detect differences was caused by a small number of participants in each group. DDI training should be a concern and added to the curriculum for improvement of the EMR training curriculum.

Emergency personnel should recognize the potential DDI in elderly patients who use multiple medications. Lin et al. reported that the prevalence of DDIs increased with age and the number of prescribed medications.[10] DDI might result from age-related physiological changes that alter pharmacokinetics and drug metabolism that could interact with other drugs. For example, renal blood flow and glomerular filtration rate were decreased in elderly patients leading to the impaired renal elimination of drugs such as lithium, insulin, atenolol, and hydrochlorothiazide.[26].

## Limitations

Our study has some limitations. First, the risk rating of DDI used in the DDI question was one of the many types of DDI risk rating classification. In our study, we used DDI risk rating classification of five levels: risk rating X (avoid combination), risk rating D (consider therapy modification), risk rating C (monitor therapy), risk rating B (no action needed), and risk rating A (no known interaction).[18] The Drug Interaction Foundation developed the “OpeRational ClassificAtion of drug interactions (ORCA) system” for classifying drug interactions into five classes: contraindicated, provisionally contraindicated, conditional, minimal risk, and no interaction.[27] In the “Drugs.com” database, there are four levels of DDI classification: major (high clinical significance, and avoid combinations), moderate (usually avoid combinations or use only under special circumstances), minor (consider an alternative drug), and unknown (no interaction information available).[28] The Drug Interaction Facts classifies three categories of severity of DDI (major, moderate, and minor), five categories of the degree of documentation (established, probable, suspected, possible, and unlikely), and a significance of 1–5 to each interaction.[29] The Micromedex Drug-Reax System classifies three categories of severity of DDI (major, moderate, and minor) and five categories of the degree of documentation (excellent, good, fair, poor, and unlikely).[29] Using railway traffic signals, Ferner et al. proposed three categories of potential harm as red (danger: do not prescribe), double amber (danger ahead: act to avoid the danger), and amber (possible harm: be aware and make patient aware of potential danger).[29, 30] The participant may be aware of DDIs but may not be familiar with the risk rating classification, influencing the score. Further study should use risk rating comparisons of multiple risk rating classifications.

Second, the participant might use a DDI checker book or application to help them answer the examination through the internet-based examination survey. Although we asked them to avoid using a DDI checker book or application during the trial, the cheating might not be eliminated. A further study may be designed by providing in-class testing to control the examination environment.

Third, we could not estimate the exact response rate of this internet-based examination survey because we disseminated an invitation message through social media as a limitation of this method. The target participant found the link to this survey and answered it themselves. We could not provide how many surveys were sent out, thus unable to calculate the exact response rate. In addition, it could not be completely assured that the respondent was the target population. However, recruitment via social media with an online survey platform was a convenient method to enroll emergency personnel widely anywhere in real-world situations. Moreover, we sent an invitation message through the specific emergency personnel social media group to ensure the respondent was the target population. In a disseminated invitation message, we emphasized that this survey was only for an emergency physician, an emergency medicine resident, or an emergency care nurse. Thus, we believed that all respondents were a target population. In further research, it may use another method, such as spreading the survey through institutional links or email accounts.

Fourth, as a subgroup analysis of an academic year of EMR, which may be due to the small number of EMR in each group. Therefore, further study might use in the formal EMR training formative examination nationwide.

Fifth, our participant was personnel in only one country. However, we recruited participants from many regions of our country who graduated from various medical and nursing schools. Medical education might vary country by country; perhaps those in other countries may have higher or lower knowledge. Thus, an international survey should be performed.

Sixth, details of the course type of clinically pharmacological or toxicological training were not recorded. This data might help determine how depth of the pharmacological knowledge in that course. Further research should concern with this issue.

## Conclusion

Factors associated with a high examination score of basic common DDI knowledge among ED personnel were EP, EMR, and the last ACLS training ≥4 years compared to ECN and no ACLS training, respectively. Factors associated with advanced DDI knowledge among ED personnel might be further investigated.

## Electronic supplementary material

Below is the link to the electronic supplementary material.

Supplementary Material 1

## Data Availability

The datasets used and/or analyzed during the current study are available from the corresponding author on reasonable request.

## Declarations

### Presentations

An abstract poster presentation was presented at the 11^th^ Asian Conference on Emergency Medicine (ACEM) 2021, held on December 17–19, 2021. The abstract was published in the Hong Kong Journal of Emergency Medicine without further editing (Asian Society for Emergency Medicine. 11^th^ Asian Conference on Emergency Medicine 2021 Conference proceedings. Hong Kong J Emerg Med. 2022;29(1_suppl):2S-87S. doi:10.1177/10249079221099636).

### Guidelines and regulations

We followed the strengthening the reporting of observational studies in epidemiology (STROBE) statement recommendations. The study was carried out in accordance with ethical guidelines of the Faculty of Medicine, Chiang Mai University.

## Abbreviations

ACLS: Advanced cardiovascular life support
ANOVA: Analysis of variance
DDI: Drug-drug interaction
ECN: Emergency care nurse
ED: Emergency department
EMR: Emergency medicine resident
EN: Emergency nurse
ENP: Emergency nurse practitioner
EP: Emergency physician
MCQ: Multiple-choice question
ORCA: OpeRational ClassificAtion of drug interactions
RN: Registered nurse.
STROBE: Strengthening the reporting of observational studies in epidemiology
95% CI: 95% confidence interval

## References

[1] de Oliveira LM, Diel J do, AC, Nunes A, da Silva Dal Pizzol T. Prevalence of drug interactions in hospitalised elderly patients: a systematic review. Eur J Hosp Pharm. 2021 Jan;28(1):4–9. 10.1136/ejhpharm-2019-002111 PMC7788180 33355278

[2] Tragni E, Casula M, Pieri V, Favato G, Marcobelli A, Trotta MG (2013). Prevalence of the prescription of potentially interacting drugs. PLoS One.

[3] Vonbach P, Dubied A, Krähenbühl S, Beer JH (2008). Prevalence of drug-drug interactions at hospital entry and during hospital stay of patients in internal medicine. Eur J Intern Med.

[4] Aljadani R, Aseeri M (2018). Prevalence of drug-drug interactions in geriatric patients at an ambulatory care pharmacy in a tertiary care teaching hospital. BMC Res Notes.

[5] Gummin DD, Mowry JB, Beuhler MC, Spyker DA, Bronstein AC, Rivers LJ, et al. 2020 Annual Report of the American Association of Poison Control Centers’ National Poison Data System (NPDS): 38^th^ Annual Report. Clin Toxicol (Phila). 2021 Dec;59(12):1282–501. 10.1080/15563650.2021.1989785 34890263

[6] Dookeeram D, Bidaisee S, Paul JF, Nunes P, Robertson P, Maharaj VR (2017). Polypharmacy and potential drug–drug interactions in emergency department patients in the Caribbean. Int J Clin Pharm.

[7] Jawaro T, Bridgeman PJ, Mele J, Wei G (2019). Descriptive study of drug-drug interactions attributed to prescriptions written upon discharge from the emergency department. Am J Emerg Med.

[8] Okoli C, Schwenk A, Radford M, Myland M, Taylor S, Darley A (2020). Polypharmacy and potential drug-drug interactions for people with HIV in the UK from the Climate-HIV database. HIV Med.

[9] Doan J, Zakrzewski-Jakubiak H, Roy J, Turgeon J, Tannenbaum C (2013). Prevalence and risk of potential cytochrome P450-mediated drug-drug interactions in older hospitalized patients with polypharmacy. Ann Pharmacother.

[10] Lin CF, Wang CY, Bai CH (2011). Polypharmacy, aging and potential drug-drug interactions in outpatients in Taiwan: a retrospective computerized screening study. Drugs Aging.

[11] Juurlink DN, Mamdani M, Kopp A, Laupacis A, Redelmeier DA (2003). Drug-drug interactions among elderly patients hospitalized for drug toxicity. JAMA.

[12] Stassen HH, Bachmann S, Bridler R, Cattapan K, Herzig D, Schneeberger A (2022). Detailing the effects of polypharmacy in psychiatry: longitudinal study of 320 patients hospitalized for depression or schizophrenia. Eur Arch Psychiatry Clin Neurosci.

[13] Bachmann P, Frahm N, Debus JL, Mashhadiakbar P, Langhorst SE, Streckenbach B (2022). Prevalence and Severity of Potential Drug-Drug Interactions in Patients with Multiple Sclerosis with and without Polypharmacy. Pharmaceutics.

[14] Pasina L, Djade CD, Nobili A, Tettamanti M, Franchi C, Salerno F (2013). Drug–drug interactions in a cohort of hospitalized elderly patients. Pharmacoepidemiol Drug Saf.

[15] Lombardi N, Crescioli G, Bettiol A, Marconi E, Vitiello A, Bonaiuti R (2018). Characterization of serious adverse drug reactions as cause of emergency department visit in children: a 5-years active pharmacovigilance study. BMC Pharmacol Toxicol.

[16] von Elm E, Altman DG, Egger M, Pocock SJ, Gøtzsche PC, Vandenbroucke JP, et al. The Strengthening the Reporting of Observational Studies in Epidemiology (STROBE) statement: guidelines for reporting observational studies. J Clin Epidemiol. 2008 Apr;61(4):344–9. 10.1016/j.jclinepi.2007.11.008 18313558

[17] Harris PA, Taylor R, Minor BL, Elliott V, Fernandez M, O’Neal L (2019). The REDCap consortium: Building an international community of software platform partners. J Biomed Inform.

[18] Lexicomp, Wolters Kluwer NV. Interactions in Lexi-drugs online [Internet]. [cited 2022 Aug 17]. Available from: http://online.lexi.com. Subscription required to view.

[19] Van WilsonVoorhis CR, Morgan BL (2007). Understanding Power and Rules of Thumb for Determining Sample Sizes. Tutor Quant Methods Psychol.

[20] Ko Y, Malone DC, Skrepnek GH, Armstrong EP, Murphy JE, Abarca J (2008). Prescribers’ knowledge of and sources of information for potential drug-drug interactions: a postal survey of US prescribers. Drug Saf.

[21] Warholak TL, Hines LE, Song MC, Gessay A, Menke JM, Sherrill D (2011). Medical, nursing, and pharmacy students’ ability to recognize potential drug-drug interactions: a comparison of healthcare professional students. J Am Acad Nurse Pract.

[22] Yuan J, Shen C, Wang C, Shen G, Han B (2021). Assessment of Physician’s Knowledge of Potential Drug-Drug Interactions: An Online Survey in China. Front Med (Lausanne).

[23] Nabovati E, Vakili-Arki H, Taherzadeh Z, Saberi MR, Abu-Hanna A, Eslami S (2017). A survey of attitudes, practices, and knowledge regarding drug-drug interactions among medical residents in Iran. Int J Clin Pharm.

[24] Moura CS, Prado NM, Belo NO, Acurcio FA (2012). Evaluation of drug-drug interaction screening software combined with pharmacist intervention. Int J Clin Pharm.

[25] Kopec KT, Vohra R, Santos C, Kazzi Z, Wong A (2020). The Global Educational Toxicology Toolkit (GETKIT): A 1-Day Course for Teaching Poisoning Essentials in Low- and Middle-Income Countries (LMIC): Course Development and Pilot Data Analysis. J Med Toxicol.

[26] Klotz U (2009). Pharmacokinetics and drug metabolism in the elderly. Drug Metab Rev.

[27] Hansten PD, Horn JR, Hazlet TK (2001). ORCA: OpeRational ClassificAtion of drug interactions. J Am Pharm Assoc (Wash).

[28] Drugs.com. Drug Interactions Checker - For Drugs, Food & Alcohol [Internet]. Drugs.com. [cited 2022 Oct 11]. Available from: https://www.drugs.com/drug_interactions.html.

[29] Aronson JK (2007). Communicating information about drug interactions. Br J Clin Pharmacol.

[30] Ferner RE, Aronson JK (2006). Communicating information about drug safety. BMJ.

