# Supplementary material for: Factors associated with a basic common drug-drug interaction knowledge among emergency department medical personnel
Source: BMC Pharmacol Toxicol. 2022 Oct 31;23:84. doi: 10.1186/s40360-022-00623-0 (PMC9620625; doi:10.1186/s40360-022-00623-0)
Supplement: Supplementary file 1 — Supplementary Material 1 [file 40360_2022_623_MOESM1_ESM.pdf]

Supplementary Material

Internet-Based Examination Survey of Research Study

Title: Factors Associated with a Basic Common Drug-Drug Interaction Knowledge Among Emergency Department Medical Personnel

Eligible participant:
-Emergency Physician (EP)
-Emergency Medicine Resident
-Emergency Care Nurse: Registered nurse (RN), Emergency nurse (EN), Emergency nurse practitioner (ENP)

Importance: This research participant is only for an emergency physician, an emergency medicine resident, or an emergency care nurse. If you are out of the scope of this profession, please send this survey to those you know.

I give my consent for information about myself to be collected by this survey.
○ Yes
○ No

Part 1 Baseline Questions

Age: ___________ years old
Sex: ○ Male ○ Female
Email (for contact to receive prize): ____________@________
Do you want to know your final score via email?
○ Yes ○ No

Profession or academic year of an emergency medicine resident
○ Emergency physician (EP)
○ First-year emergency medicine resident
○ Second-year emergency medicine resident
○ Third-year emergency medicine resident
○ Emergency care nurse: Registered nurse (RN)
○ Emergency care nurse: Emergency nurse (EN)
○ Emergency care nurse: Emergency nurse practitioner (ENP)

Academic degree
○ Bachelor's degree
○ Master's degree
○ Doctoral degree

Academic position
○ None
○ Lecturer
○ Assistant professor
○ Associate professor
○ Professor

How many years have you worked in the Emergency Department including in-training year(s)?
__________ year(s)

Have you ever passed the clinically pharmacological or toxicological training?
○ Yes ○ No

If you ever passed the clinically pharmacological or toxicological training course, how many years ago did you pass the last complete or recertification clinically pharmacological or toxicological training course? (if not applicable, please leave blank)
__________ year(s)

Do you ever pass the Advanced Cardiac Life Support (ACLS) training course?
○ Yes ○ No

If you ever pass the ACLS training course, how many years ago did you pass the last complete or recertification ACLS training course? (if not applicable, please leave blank)
__________ year(s)

Do you ever use a drug-drug interaction checker book?
○ Yes ○ No

Do you ever use drug-drug interaction checker application?
○ Yes ○ No

<u>Part 2 Examination Questions for Common Drug-Drug Interaction in the Emergency Department</u>

Instructions:

Please choose the best answer for each of the following questions. The questions are worth 1 point each. If the test is performed multiple times, the score from the first test only would be graded. Please avoid using a DDI checker book or application while answering the test. The risk rating of drug combination is based on Lexicomp® Drug Interactions database and ranges from low to high risk as follows:

-Risk rating A: no known interaction group
-Risk rating B: no action needed group
-Risk rating C: monitor therapy group
-Risk rating D: consider therapy modification group
-Risk rating X: avoid combination group

	Drug combinations	Risk rating A, B	Risk rating C, D	Risk rating X
1	Diazepam and Metronidazole	○	○	○
2	Furosemide and Amiodarone	○	○	○
3	Azithromycin and Amiodarone	○	○	○
4	Etomidate and Metronidazole	○	○	○
5	Ceftriaxone and Calcium gluconate	○	○	○
6	Diazepam and Ciprofloxacin	○	○	○
7	Heparin and Chlorpheniramine	○	○	○
8	Haloperidol and Metoclopramide	○	○	○
9	Ondansetron and Ciprofloxacin	○	○	○
10	Paracetamol and Metoclopramide	○	○	○
11	Azithromycin and Albuterol	○	○	○
12	Warfarin and Ibuprofen	○	○	○
13	Warfarin and Tramadol	○	○	○
14	Diazepam and Metoclopramide	○	○	○
15	Heparin and Hydroxyzine	○	○	○
16	Lidocaine and Ciprofloxacin	○	○	○
17	Lorazepam and Metronidazole	○	○	○
18	Diazepam and Phenytoin	○	○	○
19	Fentanyl and Lidocaine	○	○	○
20	Paracetamol and Morphine	○	○	○
21	Tramadol and Lidocaine	○	○	○
22	Heparin and Dimenhydrinate	○	○	○
23	Ondansetron and Albuterol	○	○	○

24	Potassium chloride (KCl) and Benztropine	○	○	○
25	Paracetamol and Tramadol	○	○	○
26	Tramadol and Haloperidol	○	○	○
27	Lorazepam and Chlorpheniramine	○	○	○
28	Paracetamol and Ondansetron	○	○	○
29	Potassium chloride (KCl) and Chlorpheniramine	○	○	○
30	Lidocaine and Morphine	○	○	○
31	Potassium chloride (KCl) and Phenytoin	○	○	○
32	Warfarin and Phenytoin	○	○	○
33	Azithromycin and Hydroxyzine	○	○	○
34	Potassium chloride (KCl) and Haloperidol	○	○	○
35	Furosemide and Phenytoin	○	○	○
36	Hydrocortisone and Albuterol	○	○	○
37	Potassium chloride (KCl) and Hydroxyzine	○	○	○
38	Paracetamol and Fentanyl	○	○	○
39	Warfarin and Omeprazole	○	○	○
40	Heparin and Ibuprofen	○	○	○
